# Individualized CT image-guided free-hand catheter technique: A new and reliable method for minimally invasive evacuation of basal ganglia hematoma

**DOI:** 10.3389/fnins.2022.947282

**Published:** 2022-08-25

**Authors:** Zhijie Zhao, Jinting Xiao, Jianjun Wang, Xiangjing Meng, Cuiling Li, Tao Xin, Shengjie Li

**Affiliations:** ^1^Department of Neurosurgery, the First Affiliated Hospital of Shandong First Medical University & Shandong Provincial Qianfoshan Hospital, Shandong Medicine and Health Key Laboratory of Neurosurgery, Jinan, China; ^2^Department of Medical Ultrasound, the First Affiliated Hospital of Shandong First Medical University & Shandong Provincial Qianfoshan Hospital, Shandong Medicine and Health Key Laboratory of Abdominal Medical Imaging, Jinan, China; ^3^Department of Neurosurgery, Jiangxi Provincial People's Hospital Affiliated to Nanchang University, Nanchang, China

**Keywords:** basal ganglia hematoma, CT image, stereotactic guidance, ROSA robot, minimally invasive catheter, functional outcomes

## Abstract

**Objective:**

To validate the clinical reliability of an individualized CT image-guided‘ free-hand catheter technique (CTGFC) for basal ganglia hematoma (BGH) evacuation.

**Methods:**

From January 2017 to December 2020, 58 cases of patients with BGH who underwent catheter evacuation were enrolled. The surgery was conducted using the CTGFC (*n* = 31) or stereotactic catheter technique (STC, *n* = 27). The authors evaluated the baseline characteristics, operation-related indicators, postoperative complications, hospitalization-related indicators, short-term and long-term functional outcomes, and mortality rate 1 year after surgery.

**Results:**

All patients underwent BGH evacuation under non-general anesthesia in the CTGFC group. The operative time (*p* < 0.01) and operation costs (*p* < 0.05) were significantly shorter in the CTGFC group than that in the STC group (*p* < 0.01). Comparable results were found in the catheter indwelling duration, residual hematoma volume, hematoma evacuation rate, incidence of postoperative complications, hospital ICU stay, and hospital costs between these two groups (*p* > 0.05). The duration of hospital stay was remarkably shorter in the CTGFC group than that in the STC group (*p* < 0.01). There were no differences in terms of the short-time functional outcomes score at discharge, including the Glasgow outcome scale (GOS) score, the activities of daily living (ADL) score, and the Karnofsky performance score (KPS). Moreover, comparable findings were also found in the 1-year postoperative GOS score, ADL score, KPS score, and mortality rate between these two groups.

**Conclusion:**

The simple CTGFC-assisted surgery was a safe and reliable option for BGH evacuation, especially in primary medical institutes and emergency situations with limited medical resources.

## Introduction

Spontaneous intracerebral hemorrhage (ICH) is a life-threatening global public health event with substantially high morbidity and mortality (Gross et al., 2019). Worldwide, the incidence of ICH is more than five million annually (Hanley et al., 2019). The leading cause of spontaneous ICH is deep perforating vasculopathy resulting from systemic hypertension. The most common site of spontaneous ICH is located in the deep brain structures (e.g., the basal ganglia, the cerebellum, the pons, and the thalamus) and the superficial regions of the brain (Campbell and Khatri, 2020). Deep ICH is typically associated with hypertension, whereas lobar ICH is usually related to cerebral amyloid angiopathy (Georgakis et al., 2020). The incidence of ICH, especially deep-seated ICH, tends to increase with advancing age (Hanley et al., 2019; Lioutas et al., 2020). Available data show that the mortality rate 30 days after ICH is approximately 40% and reaches approximately 60% at 1 year (Xu et al., 2018; Fallenius et al., 2019). Over two-thirds of patients survive with significant neurological dysfunction (Kuramatsu et al., 2019). The outcomes after spontaneous ICH are closely associated with age, unstable blood pressure, hematoma location, hematoma volume, midline shift, and perihematomal edema (Ironside et al., 2020; Zhang et al., 2022). Compared with the patients with lobar ICH, the patients with deep ICH suffer from even worse symptoms and poorer prognosis (Leasure et al., 2019; Ironside et al., 2020), which has generated considerable interest in deep ICH.

To date, the definite treatment strategy of deep-seated ICH remains controversial. The findings from non-surgical trials of early hemostasis and blood pressure reduction demonstrate no favorable changes in functional outcomes or mortality (Mayer et al., 2008; Anderson et al., 2013). Given the high morbidity and mortality of deep-seated ICH, surgical options have been evaluated in numerous clinical trials. Surgical approaches from macroscopic craniotomy to minimally invasive surgery (MIS) for ICH evacuation vary widely (Cordonnier et al., 2018; Gross et al., 2019). In the spontaneous supratentorial lobar intracerebral hematomas (STICH) II trial, no favorable outcomes at 6 months were found between the conventional craniotomy group and the medical management group (Mendelow et al., 2013). A possible explanation for the above finding is that functional outcomes may vary widely based on the interactions between hematoma location and volume. Consistently, the supratentorial ICH volume of more than 30 ml is commonly accepted as an ICH volume cutoff for poor outcome, trial enrollment criteria, and surgical indication (Hemphill et al., 2015; Greenberg et al., 2022). Of note, the location-specific hematoma volume threshold has recently been identified as a potential predicting factor for the severity of neurological deficit, and the optimal cut-off value to predict the poor outcome is 18 ml in basal ganglia hematoma (BGH). On the other hand, the optimal cut-off values to predict functional dependence and mortality are 21.0 and 24.0 mL, respectively, for lobar ICH (Hemphill et al., 2015). In contrast to supratentorial lobar ICH, deep-seated ICH presents a huge clinical challenge for surgical evacuation because of the necessary dissection through the white matter fiber tracts to access the lesion (Gross et al., 2019; de Oliveira Manoel, 2020). MIS for ICH evacuation is attracting considerable attention. The findings from the MISTIE (minimally invasive surgery plus alteplase for ICH evacuation) II trial showed that minimally invasive catheter (MIC) plus fibrinolytic agent (alteplase) was safe with a better functional outcome compared to standard medical treatment (Hanley et al., 2016). The favorable findings were consistent with the data presented in an updated meta-analysis of randomized controlled trials of MIS, including endoscopic surgery and stereotactic thrombolysis (Scaggiante et al., 2018). This meta-analysis suggested that select patients with supratentorial ICH benefited from MIS over other treatments, including standard medical management and craniotomy. However, in phase III of the MISTIE trial, MISTIE did not improve functional outcomes in patients with moderate to large ICH compared with standard medical management (Hanley et al., 2019).

Stereotactic techniques have been introduced into clinical practice with the aim of improving the safety and accuracy of MIS for ICH evacuation (Lefranc et al., 2015; Kellner et al., 2018; Stumpo et al., 2021). Current limitations of stereotactic techniques include time-consuming, high cost, long learning curve, and high technical requirements (D'Souza et al., 2019). Facing the opportunities and challenges in MIS assisted by modern stereotactic techniques, we cannot help wondering if neurosurgeons can perform MIS for ICH evacuation safely and efficiently in the primary medical centers without the assistance of modern stereotactic equipment and professionals in urgent conditions. In this retrospective study, we developed an individualized CTGFC for the evacuation of deep-seated BGH and validated its clinical reliability.

## Materials and methods

### Subjects

A single-center retrospective study was carried out according to the inclusion and exclusion criteria, 58 cases of patients with BGH from January 2017 to December 2020 were evaluated in the retrospective study. Patients were classified into two groups with treatment methods either CTGFC, 31 cases, or Frame-based or ROSA^®^ robot-assisted STC, 27 cases. All patients were confirmed BGH by brain CT examination. The Tada formula was used to calculate the volume of the intraparenchymal hematoma. All operations were performed by board-certified neurosurgeons with more than 8 years of experience (>100 cases), who were trained to skillfully perform both CTGFC and STC operations. To reduce selection bias caused by surgeons, individualized surgical treatment options mainly depended on the patients' family members' preferences, the patients' or equipment's conditions, and the availability of anesthesiologists, nurses, and imaging technicians at the time. The priority for treatment selection was according to the patients' family members' preferences. CTGFC operation was the treatment option when at least one of the following occurred: progressive deterioration of consciousness or neurological function before the planned STC operation, poor general anesthesia tolerability, or the shortage of available equipment and medical staff especially during the night. All enrolled patients received standard medical treatment according to the guidelines from the American Heart Association/American Stroke Association Stroke Council (Hemphill et al., 2015). We obtained all appropriate patient consent forms. All procedures performed in the study were approved by the Ethics committee.

### Inclusion and exclusion criteria

The inclusion criteria for this study were as follows: (1) age 18–80 years; (2) the main body of hematoma located in basal ganglia with or without secondary intraventricular hemorrhage; and (3) patients' families agreed to take MIC and signed the informed consent form.

The exclusion criteria were as follows: (1) unstable vital signs or brain hernia with bilateral mydriasis; (2) hematological disease; (3) ICH due to trauma, brain tumor, hemorrhagic transformation of cerebral infarction, or any other suspected secondary causes; (4) respiratory and cardiac failure; (5) recovery from cardiopulmonary resuscitation; (6) incomplete clinical data or no follow-up; and (7) automatically discharged within 3 days of hospitalization.

### Surgical technique

**Figure 1:**
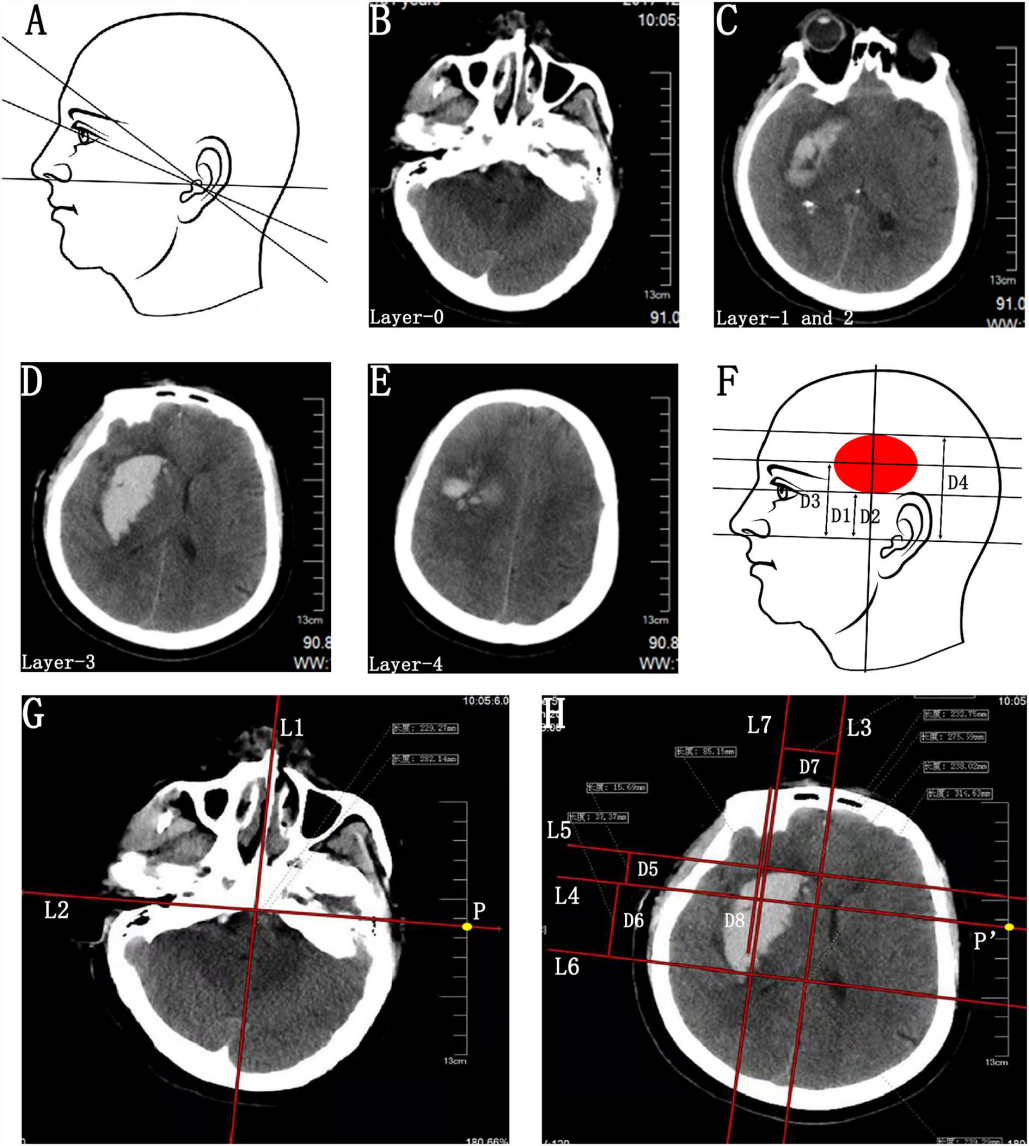
Preoperative parameter acquisition of BGH on the basis of individualized CT scan images. **(A)** Schematic diagram of various baseline planes of CT scan. **(B–E)** CT scan image passing through the external auditory canal (Layer-0) **(B)**, eye's lenses (Layer-1) **(C)**, bottommost (Layer-2) **(C)**, largest (Layer-3) **(D)**, and uppermost (Layer-4) **(E)** layers of BGH. Layer-1 and Layer-2 exactly coincided in this case. **(F)** Schematic diagram of hatch spacing distance between Layer-0 and Layer-1 (D1), Layer-2 (D2), Layer-3 (D3), and Layer-4 (D4). D1 and D2 were the same in this case. **(G)** On Layer-0, a midsagittal line (L1) and its perpendicular line (L2) passing through the bilateral external auditory canal were drawn, making sure that the L2 was long enough to intersect with the CT scale at point P. The point P was defined as the sagittal projection point of the external auditory canal. **(H)** On Layer-3, a midsagittal line (L3) and its perpendicular line (L4) passing through the point P′ were drawn according to the same method as above, making sure that points P and P′ were at the same position on the CT scale. The point P′ was defined as the sagittal projection point of the external auditory canal on this CT scan layer and L4 was identified as the reference line of the bilateral external auditory canal based on the actual CT baseline plane. Two parallel lines (L5 and L6) of the L4 were drawn passing through the anterior and posterior border of hematoma, respectively. Then, the distance between the parallel lines (L5-L4 and L6-L4) was measured and defined as D5 and D6. A line (L7) parallel to the L3 was drawn passing through the center of BGH. The distance between L3 and L7 was D7. The D7 was recognized as the distance of the planned puncture point lateral to the L3. Due to morphological heterogeneity of hematoma, the planned penetration depth (D8) of the catheter was defined as the distance along the line L7 from the head surface to the midpoint or anterior 2/3 point of the hematoma. BGH, basal ganglia hematoma.

**Figure 2:**
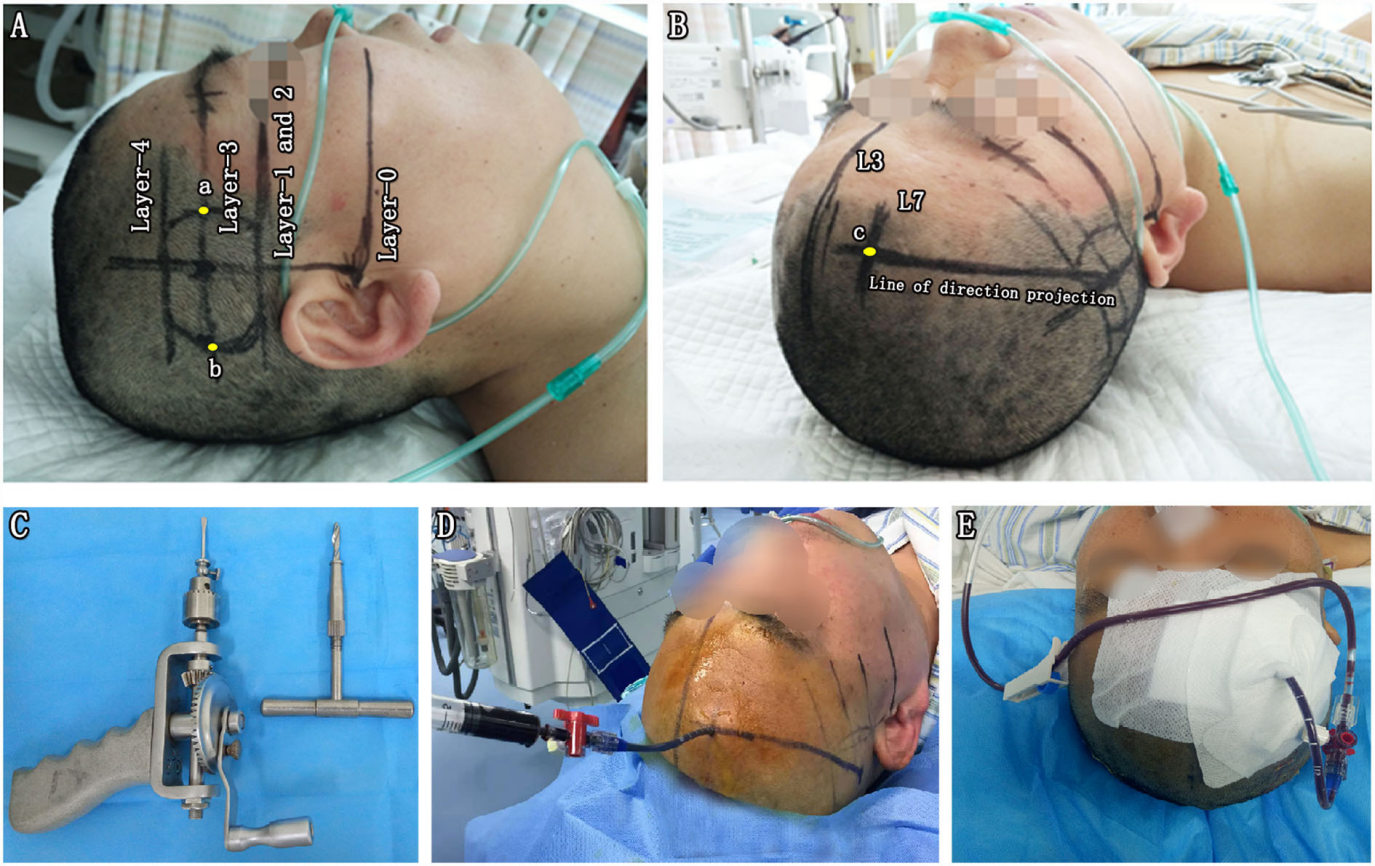
Parameter reproduction on a patient's head and free-hand catheter technique. **(A)** Reproducing the actual baseline plane of the CT scan on the patient's head and outlining the sagittal projection of the hematoma border. With the help of a triangle ruler, two parallel baselines were drawn passing through the external auditory canal (Layer-0) and eye's lenses (Layer-1) with the distance of D1. Next, the CT scan axis was drawn through the external auditory. The projection lines of the bottommost (Layer-2), largest (Layer-3), and uppermost (Layer-4) layers of BGH were depicted on the ipsilateral temporal skin, according to the distance (D2, D3, and D4) to Layer-0. Layer-1 and Layer-2 exactly coincided in this case. On the projection line of Layer-3, the projection points of the anterior (point a) and posterior (point b) borders of the hematoma were marked according to D5 and D6. **(B)** The midsagittal line L3 and paramedian sagittal line L7 were drawn with the reference of D7. The puncture point (point c) was located at the forehead area between 2 cm above eyebrow arch and coronal suture away from the vascular and functional distribution of brain tissue. A marking line of the puncture direction was drawn from point c to the center of the temporal skin projection area of the BGH. **(C)** A hand drill and a reamer. **(D)** Aspiration with a 5-mL syringe. **(E)** Hematoma drainage. BGH, basal ganglia hematoma.

For the CTGFC group, the detailed protocol for preoperative parameter acquisition of BGH and parameter reproduction on a patient's head is illustrated in Figures 1, 2.

Step 1. Identification of the actual baseline plane. (1) Identification of the images passing through the external auditory canal (Layer-0; Figure 1B) and eye's lenses (Layer-1; Figure 1C). (2) Calculating the hatch spacing distance (D1) between Layer-0 and Layer-1 according to the layer thickness and the layer number intervals (Figure 1F).

Step 2. Parameter acquisition of the upper, lower, anterior, and posterior bounds of the BGH. **(1)** Identification of the CT scan images passing through the bottommost (Layer-2; Figure 1C), largest (Layer-3, Figure 1D), and uppermost (Layer-4; Figure 1E) layers of BGH, then calculate the hatch spacing distance (D2, D3, and D4) between layer-0 and layer-2, layer-3, and layer-4, respectively (Figure 1F). Layer-1 and Layer-2 exactly coincided in this case. (2) The images of Layer-0 and Layer-3 were chosen as the target layers for further parameter acquisition. On Layer-0, a midsagittal line (L1) and its perpendicular line (L2) passing through the bilateral external auditory canal were drawn, making sure that the L2 was long enough to intersect with the CT scale at point P (Figure 1G). The point P was the sagittal projection point of the external auditory canal. (3) On Layer-3, a midsagittal line (L3) and its perpendicular line (L4) passing through the point P′ were drawn according to the same method as above, making sure that points P and P′ were at the same position on the CT scale (Figure 1H). The point P′was the sagittal projection point of the external auditory canal on this CT scan layer. Since all images were axial images obtained parallel to the actual baseline plane, L4 was identified as the reference line of the bilateral external auditory canal based on the actual baseline plane. (4) Two parallel lines (L5 and L6) of the L4 were drawn passing through the anterior and posterior border of hematoma, respectively (Figure 1H). Then, the distance between the parallel lines (L5–L4 and L6–L4) was measured and defined as D5 and D6 (Figure 1H).

Step 3. Parameter acquisition of the penetration depth of catheter. On Layer-3, a line (L7) parallel to the L3 was drawn passing through the center of BGH. The distance between L3 and L7 was D7. The D7 was recognized as the distance of the planned puncture point lateral to the L3. Due to morphological heterogeneity of hematoma, the planned penetration depth (D8) was defined as the distance along the line L7 from the head surface to the midpoint or anterior 2/3 point of the hematoma (Figure 1H).

Step 4. Reproducing the preoperative parameters on the surface of the patient's head to outline the sagittal projection of the hematoma border and planned puncture direction. (1) Reproducing the actual baseline plane of the CT scan on the patient's head. With the help of a triangle ruler, two parallel baselines were drawn passing through the external auditory canal (Layer-0) and eye's lenses (Layer-1) with the distance of D1. Next, the CT scan axis was drawn through the external auditory (Figure 2A). (2) Since all CT scan layers were axial layers parallel to the actual baseline plane, the projection lines of the bottommost (Layer-2), largest (Layer-3), and uppermost (Layer-4) layers of BGH were depicted on the ipsilateral temporal skin, according to the distance (D2, D3, and D4) to Layer-0 (Figure 2A). Layer-1 and Layer-2 exactly coincided in this case. (3) On the projection line of Layer-3, the projection points of the anterior (point a) and posterior (point b) borders of the hematoma were marked according to D5 and D6 (Figure 2A). Sagittal projection of the BGH was depicted on the ipsilateral temporal skin through the above-mentioned method (Figure 1H). (4) The midsagittal line L3 and paramedian sagittal line L7 were drawn with the reference of D7 (Figure 2B). (5) The puncture point (point C) was located at the forehead area between 2 cm above the eyebrow arch and coronal suture away from the vascular and functional distribution of brain tissue (Figure 2B). A marking line of the puncture direction was drawn from point C to the center of the temporal skin projection area of the BGH (Figure 2B).

Under non-general anesthesia at the bedside or in the operating room, the standard operating procedures in the CTGFC group were performed as follows. A 4-mm-diameter hole was drilled at point C with a hand drill and a reamer (SHINVA, Zibo, Shandong, China; Figure 2C). Through the above skull hole, we placed a soft catheter into the hematoma along the L7 guided by the marking line. The aspiration was performed with a 5-ml syringe until no fluid component was in the syringe or until the first resistance (Figures 2D,E). Postoperative CT in 24 h was performed to confirm the positioning of the catheter and the residual volume of hematoma.

The STC operation was done under non-general or general anesthesia in the operating room. The procedures were performed in a standard manner as follows. A soft catheter was placed into the center or two-thirds length of the long axis of hematoma through a 12-mm-diameter burr hole using the stereotactic frame or ROSA robot (Medtech, Montpellier, France) according to the preoperative CT scan. The entry point and puncture path should avoid functional domains and the main blood vessels.

Hemorrhage clot aspiration: If the residual volume was >15 ml or bigger than half of the initial volume, the intraclot administration of thrombolysis (urokinase) was performed in a dose of 2–5 × 10^4^ IU one or two times a day. The follow-up CT scans were performed as clinically indicated and before catheter removal.

### Follow-up, short-term, and long-term outcome assessment

The baseline characteristics, operation-related indicators, incidence of postoperative complications, and hospitalization-related indicators were compared. The short-term functional outcomes at discharge and long-term functional outcomes at 1 year after surgery, including GOS, ADL, and KPS score, were assessed. All the patients were followed up by telephone for long-term functional outcomes assessment.

### Statistical methods

Statistical analysis was performed by SPSS Statistics (version 26.0; SPSS Inc, Chicago, IL, USA). Categorical variables were analyzed by Fisher's exact test or chi-squared test with continuity correction, as appropriate. After confirmation of the distribution of continuous variables, data were expressed as the mean ± SD. Independent samples Student's *t*-test was used for comparison of continuous variables. A *p*-value of <0.05 was considered statistically significant.

## Results

### General results

Fifty-eight cases of patients were evaluated in this study: 31 in the CTGFC group and 27 in the STC group. There was no significant difference in baseline characteristics between the CTGFC and STC evacuation groups, with the exceptions of history of hypertension (30/31 vs. 20/27, *p* < 0.05), history of antiplatelet or anticoagulant medication (11/31 vs. 2/27, *p* < 0.05), and preoperative hematoma volume (37.52 ± 16.18 ml vs. 27.97 ± 14.32 ml, *p* < 0.05; Table 1).

**Table 1:** Characteristics of the patients at baseline.

**Characteristic**	**CT image-guided free-hand catheter group** **(*n* = 31)**	**Stereotactic catheter group** **(*n* = 27)**	**P Value**
Age (years)	56.64 ± 11.26	54.52 ± 10.40	0.965
Sex (male/female)	16/15	14/13	0.986
Preoperative hematoma volume (ml)	37.52 ± 16.18	27.97 ± 14.32	0.021
Glasgow coma scale score	10.81 ± 2.76	11.15 ± 2.85	0.645
Side (left/right)	9/22	13/14	0.134
Hematoma extending into the ventricle (yes/no)	15/16	8/19	0.145
Past hemorrhagic stroke (yes/no)	4/27	1/26	0.438
Kidney disease (yes/no)	6/25	1/26	0.155
Hypertension (yes/no)	30/1	20/7	0.034
diabetes mellitus (yes/no)	2/29	2/25	1.000
Past cardiovascular disease (yes/no)	4/27	2/25	0.800
Antiplatelet or anticoagulant agent (yes/no)	11/20	2/25	0.025
Smoking (yes/no)	8/23	6/21	0.750
Drinking (yes/no)	11/20	9/18	0.864

### Operation-related indicators, incidence of postoperative complications, and hospitalization-related indicators

**Figure 3:**
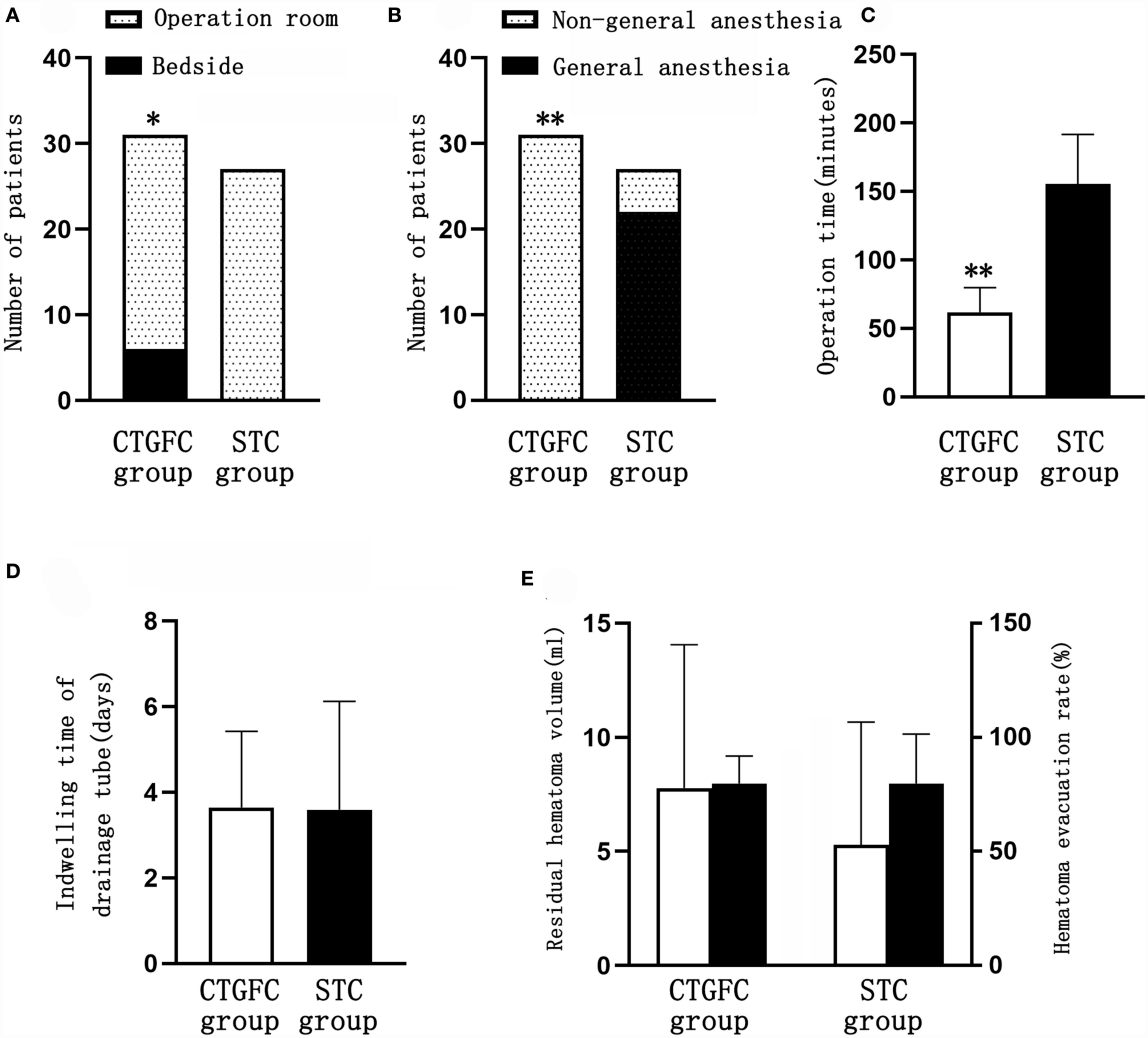
Comparison of operation-related indicators between the two groups. **(A)** Site of surgery. **(B)** Mode of anesthesia. **(C)** Operation time. **(D)** Catheter indwelling duration. **(E)** Residual hematoma volume and hematoma evacuation rate. *p < 0.05 and **p < 0.01 vs. the stereotaxic surgery group. CTGFC, CT image-guided free-hand catheter technique; STC, stereotactic catheter technique.

**Figure 4:**
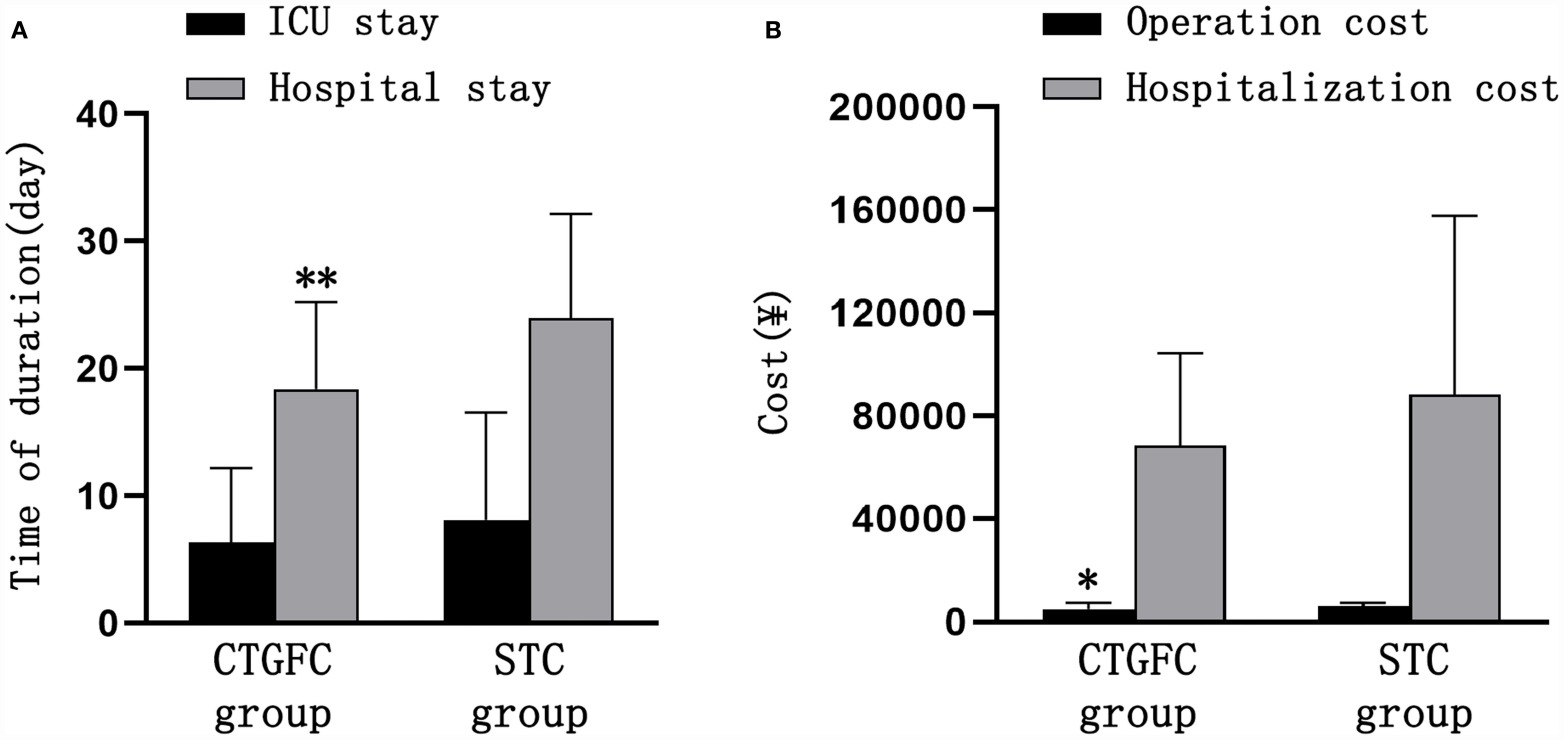
Comparison of hospitalization-related indicators between the two groups. **(A)** Length of ICU stay and hospital stay. **(B)** Operation and hospitalization costs. *p < 0.05 and **p < 0.01 vs. the stereotaxic surgery group. CTGFC, CT image-guided free-hand catheter technique; STC, stereotactic catheter technique.

Six patients underwent MIC at the bedside in the CTGFC group in contrast to none in the STC group (6/31 vs. 0/27, *p* < 0.05; Figure 3A). All the operations were performed under non-general anesthesia in the CTGFC group, significantly different from that in the STC group (31/31 vs. 5/27, *p* < 0.01; Figure 3B). The operation duration was significantly shorter in the CTGFC group than that in the STC group (61.87 ± 17.77 min vs. 155.26 ± 36.37 min, *p* < 0.01, Figure 3C). The catheter indwelling duration (3.65 ± 1.78 day vs. 3.59 ± 2.53 day, *p* = 0.927, Figure 3D), residual hematoma volume (7.78 ± 6.27 ml vs. 5.29 ± 5.37 ml, *p* = 0.112; Figure 3E), and hematoma evacuation rate (79.84 ± 11.97% vs. 79.84 ± 21.62%, *p* = 1.00; Figure 3E) of both groups were similar. The operation costs were lower in the CTGFC group than that in the STC group (4822.64 ± 2374.97¥ vs. 5908.98 ± 1391.61¥, *p* < 0.05; Figure 4B). No significant difference was observed between the two groups in the incidence of postoperative complications (Table 2). The length of ICU stay (6.35 ± 5.84 day vs. 8.11 ± 8.44 day, *p* = 0.365; Figure 4A) and hospital costs (68375.86 ± 35790.99¥ vs. 88231.91 ± 69413.27¥, *p* = 0.168; Figure 4B) in the CTGFC group seemed lower than that in the STC group but without significant difference. The length of hospital stay was shorter in the CTGFC group than that in the STC group (18.39 ± 6.81 days vs. 23.96 ± 8.19 days, *p* < 0.01; Figure 4A).

**Table 2:** Comparison of postoperative complications between the two groups.

**Variable**	**CT image-guided free-hand catheter group** **(*n* = 31)**	**Stereotactic catheter group** **(*n* = 27)**	**P Value**
Intracranial infection (n/%)	2/6.45	2/7.40	1.000
Pulmonary infection (n/%)	4/12.9	4/14.8	1.000
Blood flow infection (n/%)	1/3.2	0	1.000
Operation-related hematoma expansion (*n*/%)	1/3.2	1/3.7	1.000
Epilepsy (*n*/%)	1/3.2	0	1.000
Gastrointestinal bleeding (*n*/%)	0	1/3.7	0.466

### Short-term and long-term functional outcomes

**Figure 5:**
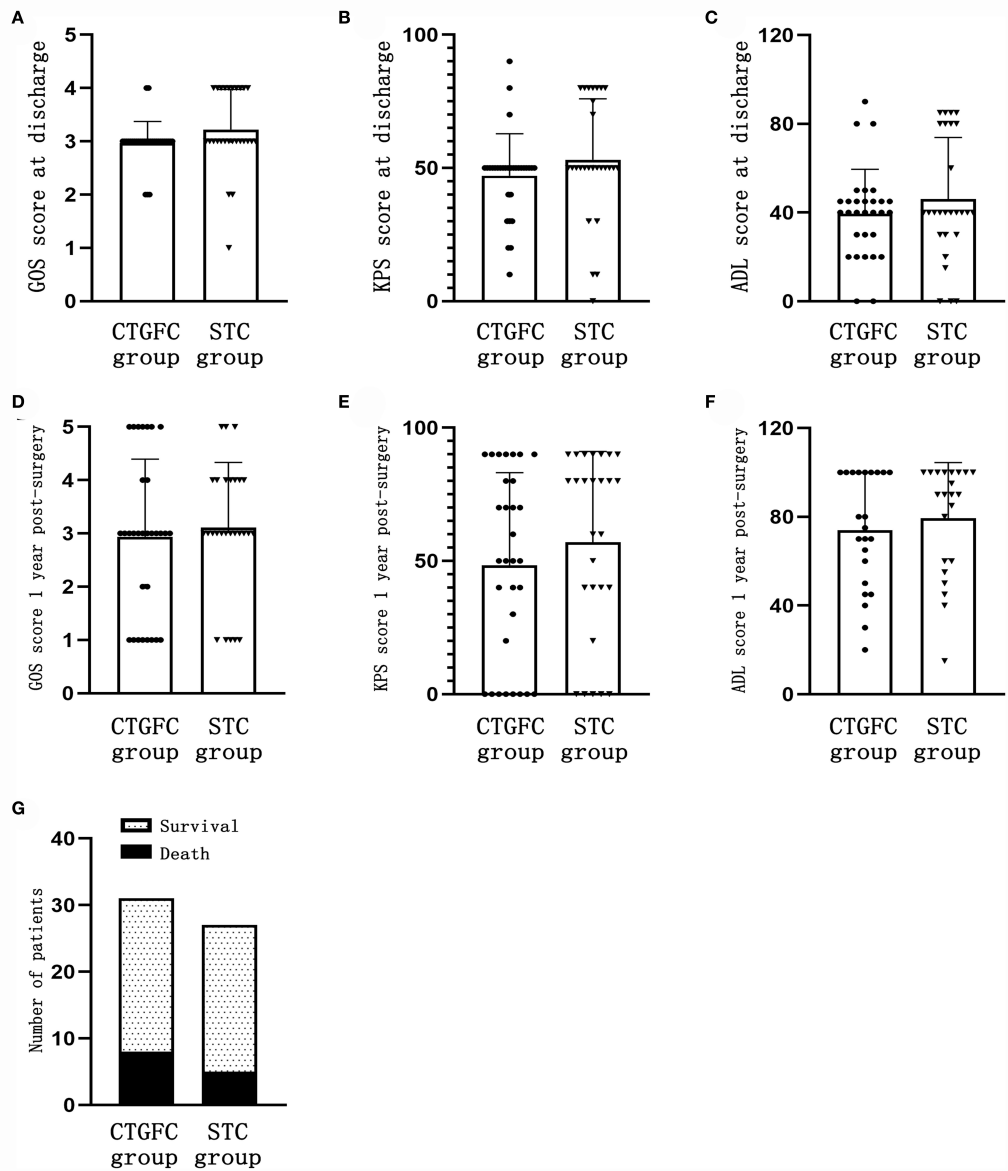
Short-time and long-term functional outcomes. **(A)** GOS score at discharge. **(B)** KPS score at discharge. **(C)** ADL score at discharge. **(D)** GOS score 1 year after the operation. **(E)** KPS score 1 year after the operation. **(F)** ADL score 1 year after the operation. **(G)** Mortality rate 1 year after the operation. CTGFC, CT image-guided free-hand catheter technique; STC, stereotactic catheter technique; GOS, Glasgow outcome scale; KPS, Karnofsky performance score; ADL, activities of daily living.

The short-term functional outcomes at the discharge of both groups were similar, including the GOS (2.97 ± 0.41 vs. 3.22 ± 0.75, *p* = 0.124; Figure 5A), KPS (47.09 ± 15.75 vs. 53.15 ± 22.83, *p* = 0.240; Figure 5B), and ADL (38.22 ± 20.96 vs. 47.88 ± 26.69, *p* = 0.132; Figure 5C). The long-term functional outcomes at 1 year, *via* telephone follow-up, were of no significant difference, which was the GOS (2.93 ± 1.46 vs. 3.11 ± 1.22, *p* = 0.620; Figure 5D), KPS (48.39 ± 34.75 vs. 57.03 ± 34.06, *p* = 0.344; Figure 5E), and ADL (73.91 ± 25.89 vs. 79.32 ± 25.08, *p* = 0.480; Figure 5F). We found no significant difference in mortality rate 1 year after operation between these two groups (8/31, 25.8% vs. 5/27, 18.5%, *p* = 0.507; Figure 5G).

## Discussion

Treatment options for patients with supratentorial ICH, especially deep-seated ICH remain controversial. Furthermore, the minimally invasive techniques, including free-hand catheter, stereotactic catheter, and endoscopic aspiration, have not been recommended as routine surgical options because of the lack of high-quality evidence (Hemphill et al., 2015; de Oliveira Manoel, 2020). Herein, we developed an individualized CTGFC technique for the evacuation of deep-seated BGH and validated its clinical reliability. Despite some differences existing in baseline characteristics, such as the history of hypertension, history of hypertension and antithrombotic medication, and preoperative hematoma volume, which had an adverse effect on the CTGFC group, there were still comparable results found in hematoma clearance efficiency, complications rate, short-term and long-term neurological outcomes, and 12-month survival rate between the CTGFC group and the STC group.

As reported in a large retrospective study involving 141,311 patients with ICH, prior use of antithrombotic medication was associated with higher in-hospital mortality (Inohara et al., 2018). Although prior use of antithrombotic medication is not an absolute contraindication to surgical treatment for ICH patients, surgeons are often reluctant to perform surgical hematoma evacuation in the acute setting, because of the potentially impaired systemic coagulation cascade and high risk of perioperative bleeding. To date, researchers have not fully revealed the benefit-risk profile of continuing or withholding surgical treatment in ICH patients with prior use of antithrombotic medication. This clinical dilemma draws attention to MIS. In this study, the administration of antithrombotic drugs was not defined as an exclusion criterion. Most patients with a history of antithrombotic medication received the CTGFC operation because of the smaller surgery-related injury compared with the STC operation. Although the priority for MIC operations selected in this study was the patients' family members' preferences, our team members tended to perform the CTGFC operation when at least one of the following occurred: progressive deterioration of consciousness or neurological function before the planned STC operation, poor general anesthesia tolerability, or the shortage of available equipment and medical staff especially during the night, which may be the plausible explanations for the reported differences in baseline characteristics. Despite a higher history of antithrombotic medication in the CTGFC group compared with that in the STC group, there was no evidence of an increased risk of operation-related new intracranial hemorrhage or expansion of primary hematoma and 1-year mortality rate. Our findings suggested that MIC operations, especially the CTGFC operation, may be wise treatment options for patients receiving long-term antithrombotic medication. Further large prospective studies are required to determine the safety and efficacy of minimal invasive hematoma evacuation in the special population of BGH patients.

Despite the high disability and mortality rates of supratentorial ICH, there are no evidence-based first-line treatment options (Mendelow et al., 2005, 2013; Hemphill et al., 2015; Qureshi et al., 2020). Large randomized trials showed that patients with supratentorial ICH failed to benefit from routine craniotomy in overall favorable outcomes or mortality at 6 months compared with conservative treatment (Mendelow et al., 2005, 2013). As a promising treatment option to improve functional outcomes of deep ICH, minimally invasive ICH evacuation has become the focus of multiple clinical trials to determine hematoma evacuation efficiency and baseline characteristics of ICH patients benefiting from it. A meta-analysis of five randomized trials and nine prospective studies involving 2,466 patients with spontaneous supratentorial ICH found the superiority of MIS in reducing rates of rebleeding and mortality, and increasing better recovery compared with conventional craniotomy (Xia et al., 2018). In a 2019 prospective multicenter randomized clinical trial, MISTIE first revealed benefit thresholds (residual hematoma volume ≤30 ml or >53% hematoma volume reduction) by MIS in decreasing mortality and improving functional outcomes (Awad et al., 2019). Favorable functional outcomes at 1 year were achievable if residual hematoma volume was ≤15 ml or ≥70% hematoma clearance rate. Only 58% of patients in the MISTIE group met the surgical aim (residual hematoma volume ≤15 ml), which potentially explained the worse functional outcomes in the MISTIE group. Notably, in this study, the average residual hematoma volume and hematoma clearance rate were 7.78 ml, 79.84% in the CTGFC group and 5.29 ml, 79.84% in the STC group, respectively, far beyond the reported threshold. The findings from the MISTIE-II trial involving 96 ICH patients revealed that MIC plus alteplase was safe but did not reduce 30-day mortality (Hanley et al., 2016). In phase III of the MISTIE trial involving 78 hospitals worldwide, in spite of reducing 180-day mortality and 30-day incidence of serious adverse events, MIS showed no superiority in improving 1-year functional outcomes in patients with moderate to large ICH compared with standard medical care (Hanley et al., 2019). The possible explanation was that the proportion of patients with lobar ICH was as high as 42% in the standard medical care group, which was recognized as a baseline variable associated with a better prognosis (Mendelow et al., 2013). MIC followed by thrombolysis has become a widely popular practice, especially in China because of the short operative time and minor invasion. As reported in this study, our findings showed the mortality rate 1 year after MIC surgery was 25.8% (8/31) in the CTGFC group and 18.5% (5/27) in the STC group, which was much lower than the reported mortality rate at 6 months in the conventional craniotomy group (36%) and the conservative treatment group (37%) (Mendelow et al., 2005).

Definite recommendations regarding the techniques of MIC for BGH evacuation are not available in existing international guidelines due to a lack of high-quality supporting evidence (Hemphill et al., 2015). The balance of risk and benefit from CTGFC for BGH remains unclear. Precise positioning is the cornerstone of surgical intervention of intracranial lesions (Kellner et al., 2018) and the STC techniques are the golden standard for accurate positioning of intracranial lesions (Lefranc et al., 2015; Kellner et al., 2018; Stumpo et al., 2021). Despite the unquestionable accuracy and widespread applications, STC techniques still have room for improvement. First, these techniques are time-consuming due to the need for transport of patients to the operation room, preparation of stereotactic or ROSA robot-guided navigation, intubation, sedation, and general anesthesia (D'Souza et al., 2019). The complicated technique and instrument requirements may make it challenging in adopting this approach at low-level medical institutes. Second, all of these techniques require an operating room with specialized equipment and human resources of coordination with anesthesiologists, nurses, and imaging technicians, which are not always available around the clock. Long-time general anesthesia is correlated with an imbalance of the immune system, postoperative pneumonia, worse neurological outcomes, and increased mortality (Liu et al., 2011; Abou-Chebl et al., 2015). Moreover, the higher stereotactic technique-related costs mean a huge burden for low-income families. Meanwhile, there is limited evidence to support CTGFC techniques. Due to the main limitations of STC techniques and the devastating course of ICH, we evaluated the clinical feasibility of an individualized CTGFC technique in emergent BGH evacuation surgery. In contrast to the STC technique, the CTGFC technique for deep-seated BGH evacuation can be easily achieved without high reliance on medical equipment and human resources. Another advantage of the CTGFC technique is less time-consuming. The operation duration of the CTGFC technique was significantly shorter than that of the STC technique (60.26 ± 20.07 min vs. 155.26 ± 36.37 min). The CTGFC technique is not dependent on general anesthesia, less invasive than the STC technique and routine craniotomy, which also reduces time consumption and avoids the side effect of general anesthesia. This advantage is crucial for severe patients with massive ICH, who need immediate surgical intervention, and for those with severe comorbidities and an inability to tolerate general anesthesia. Besides, the CTGFC technique can also be smoothly performed at the bedside without strict professional and equipment requirements when patients are in urgent situations. The above findings validated the technical safety and feasibility of the CTGFC technique in the evacuation of deep-seated BGH. Together with the low cost of CTGFC, it is a promising cost-effective technique for positioning and evacuation of BGH, especially in low- and middle-income countries with limited medical resources.

### Limitations

Several limitations of this study deserve mention. First, this was a single-center retrospective study with a relatively small sample size instead of a prospective randomized study. There might have been surgical technique and data selection bias. Some imbalances in baseline characteristics including the history of hypertension, history of antiplatelet or anticoagulant medication, and preoperative hematoma volume may affect the generalizability of our findings. More efforts should focus on the investigation of variables and a special population that might affect the therapeutic efficacy of minimal invasive ICH evacuation. Second, the Tada formula is a simple, inaccurate measurement of volume with a certain range of estimation errors. Finally, cerebrovascular imaging examination was not routinely applied before the operation to rule out vascular malformations (e.g., arteriovenous malformations and cavernous malformations) in patients with BGH.

## Conclusion

In this retrospective study, comparable results have been found in hematoma clearance efficiency, complications rate, short-term and long-term neurological outcome, and 12-month survival rate between the CTGFC group and the STC group. Furthermore, the reported CTGFC is a reliable complement to the minimal invasive evacuation of BGH, characterized by timesaving and low-cost without complicated requirements of long-time general anesthesia, stereotactic navigation instrument, and coordination of human resources. Further large prospective studies are required to determine the generalizability of our findings to other centers and assess their cost-effectiveness on functional outcomes.

## Data availability statement

The original contributions presented in the study are included in the article/supplementary material, further inquiries can be directed to the corresponding author/s.

## Ethics statement

The studies involving human participants were reviewed and approved by the Ethics Committee of the First Affiliated Hospital of Shandong First Medical University & Shandong Provincial Qianfoshan Hospital. The patients/participants provided their written informed consent to participate in this study.

## Author contributions

TX and SL contributed to the study design. ZZ, SL, JW, XM, and CL contributed to the study performance. ZZ and JX analyzed the data and wrote the manuscript draft. All authors contributed to the article and approved the submitted version.

## Funding

This study was supported by the National Natural Science Foundation of China (Grant No. 82001318), the Shandong Provincial Natural Science Foundation (Grant No. ZR2020QH119), the Development Project of Science and Technology for Youth Innovation Teams in Colleges and Universities of Shandong Province (2021KJ095), the Special Funding for Qilu Sanitation and Health Outstanding Young Talent Cultivation Project to SL, and the Academic Promotion Programme of Shandong First Medical University (2019LJ005).

## Conflict of interest

The authors declare that the research was conducted in the absence of any commercial or financial relationships that could be construed as a potential conflict of interest.

## Publisher's note

All claims expressed in this article are solely those of the authors and do not necessarily represent those of their affiliated organizations, or those of the publisher, the editors and the reviewers. Any product that may be evaluated in this article, or claim that may be made by its manufacturer, is not guaranteed or endorsed by the publisher.

## References

[B1] Abou-Chebl A. Yeatts S. D. Yan B. Cockroft K. Goyal M. Jovin T.. (2015). Impact of general anesthesia on safety and outcomes in the endovascular arm of interventional management of stroke (IMS) III trial. Stroke 46, 2142–2148. 10.1161/STROKEAHA.115.008761 26138125 PMC4519363

[B2] Anderson C. S. Heeley E. Huang Y. Wang J. Stapf C. Delcourt C.. (2013). Rapid blood-pressure lowering in patients with acute intracerebral hemorrhage. N. Engl. J. Med. 368, 2355–2365. 10.1056/NEJMoa1214609 23713578

[B3] Awad I. A. Polster S. P. Carrion-Penagos J. Thompson R. E. Cao Y. Stadnik A.. (2019). Surgical performance determines functional outcome benefit in the minimally invasive surgery plus recombinant tissue plasminogen activator for intracerebral hemorrhage evacuation (MISTIE) procedure. Neurosurgery 84, 1157–1168. 10.1093/neuros/nyz077 30891610 PMC6537634

[B4] Campbell B. C. V. Khatri P. (2020). Stroke. Lancet 396, 129–142. 10.1016/S0140-6736(20)31179-X 32653056

[B5] Cordonnier C. Demchuk A. Ziai W. Anderson C. S. (2018). Intracerebral haemorrhage: current approaches to acute management. Lancet 392, 1257–1268. 10.1016/S0140-6736(18)31878-6 30319113

[B6] de Oliveira Manoel A. L. (2020). Surgery for spontaneous intracerebral hemorrhage. Crit. Care 24, 45. 10.1186/s13054-020-2749-2 32033578 PMC7006102

[B7] D'Souza M. Gendreau J. Feng A. Kim L. H. Ho A. L. Veeravagu A. (2019). Robotic-assisted spine surgery: history, efficacy, cost, and future trends. Robot. Surg. 6, 9–23. 10.2147/RSRR.S190720 31807602 PMC6844237

[B8] Fallenius M. Skrifvars M. B. Reinikainen M. Bendel S. Curtze S. Sibolt G.. (2019). Spontaneous intracerebral hemorrhage. Stroke 50, 2336–2343. 10.1161/STROKEAHA.118.024560 31311464

[B9] Georgakis M. K. Malik R. Anderson C. D. Parhofer K. G. Hopewell J. C. Dichgans M. (2020). Genetic determinants of blood lipids and cerebral small vessel disease: role of high-density lipoprotein cholesterol. Brain 143, 597–610. 10.1093/brain/awz413 31968102 PMC7009571

[B10] Greenberg S. M. Ziai W. C. Cordonnier C. Dowlatshahi D. Francis B. Goldstein J. N.. (2022). 2022 guideline for the management of patients with spontaneous intracerebral hemorrhage: a guideline from the American Heart Association/American Stroke Association. Stroke 53, e282-e361. 10.1161/STR.0000000000000407 35579034

[B11] Gross B. A. Jankowitz B. T. Friedlander R. M. (2019). Cerebral intraparenchymal hemorrhage: a review. JAMA 321, 1295–1303. 10.1001/jama.2019.2413 30938800

[B12] Hanley D. F. Thompson R. E. Muschelli J. Rosenblum M. McBee N. Lane K.. (2016). Safety and efficacy of minimally invasive surgery plus alteplase in intracerebral haemorrhage evacuation (MISTIE): a randomised, controlled, open-label, phase 2 trial. Lancet Neurol. 15, 1228–1237. 10.1016/S1474-4422(16)30234-4 27751554 PMC5154627

[B13] Hanley D. F. Thompson R. E. Rosenblum M. Yenokyan G. Lane K. McBee N.. (2019). Efficacy and safety of minimally invasive surgery with thrombolysis in intracerebral haemorrhage evacuation (MISTIE III): a randomised, controlled, open-label, blinded endpoint phase 3 trial. Lancet 393, 1021–1032. 10.1016/S0140-6736(19)30195-3 30739747 PMC6894906

[B14] Hemphill J. C. 3rd Greenberg, S. M. Anderson C. S. Becker K. Bendok B. R. Cushman M.. (2015). Guidelines for the management of spontaneous intracerebral hemorrhage: a guideline for healthcare professionals from the American Heart Association/American Stroke Association. Stroke 46, 2032–2060. 10.1161/STR.0000000000000069 26022637

[B15] Inohara T. Xian Y. Liang L. Matsouaka R. A. Saver J. L. Smith E. E.. (2018). Association of intracerebral hemorrhage among patients taking non-vitamin K antagonist vs vitamin k antagonist oral anticoagulants with in-hospital mortality. JAMA 319, 463–473. 10.1001/jama.2017.21917 29372247 PMC5839299

[B16] Ironside N. Chen C. J. Dreyer V. Christophe B. Buell T. J. Connolly E. S. (2020). Location-specific differences in hematoma volume predict outcomes in patients with spontaneous intracerebral hemorrhage. Int. J. Stroke 15, 90–102. 10.1177/1747493019830589 30747614

[B17] Kellner C. P. Chartrain A. G. Nistal D. A. Scaggiante J. Hom D. Ghatan S.. (2018). The stereotactic intracerebral hemorrhage underwater blood aspiration (SCUBA) technique for minimally invasive endoscopic intracerebral hemorrhage evacuation. J. Neurointerv. Surg. 10, 771–776. 10.1136/neurintsurg-2017-013719 29572265 PMC6278654

[B18] Kuramatsu J. B. Sembill J. A. Huttner H. B. (2019). Reversal of oral anticoagulation in patients with acute intracerebral hemorrhage. Crit. Care 23, 206. 10.1186/s13054-019-2492-8 31171018 PMC6555738

[B19] Leasure A. C. Sheth K. N. Comeau M. Aldridge C. Worrall B. B. Vashkevich A.. (2019). Identification and validation of hematoma volume cutoffs in spontaneous, supratentorial deep intracerebral hemorrhage. Stroke 50, 2044–2049. 10.1161/STROKEAHA.118.023851 31238829 PMC6646054

[B20] Lefranc M. Capel C. Pruvot-Occean A. S. Fichten A. Desenclos C. Toussaint P.. (2015). Frameless robotic stereotactic biopsies: a consecutive series of 100 cases. J. Neurosurg. 122, 342–352. 10.3171/2014.9.JNS14107 25380111

[B21] Lioutas V. A. Beiser A. S. Aparicio H. J. Himali J. J. Selim M. H. Romero J. R.. (2020). Assessment of incidence and risk factors of intracerebral hemorrhage among participants in the framingham heart study between 1948 and 2016. JAMA Neurol. 77, 1252–1260. 10.1001/jamaneurol.2020.1512 32511690 PMC7281354

[B22] Liu S. Wang B. Li S. Zhou Y. An L. Wang Y.. (2011). Immune cell populations decrease during craniotomy under general anesthesia. Anesth. Analg. 113, 572–577. 10.1213/ANE.0b013e3182278237 21813628

[B23] Mayer S. A. Brun N. C. Begtrup K. Broderick J. Davis S. Diringer M. N.. (2008). Efficacy and safety of recombinant activated factor VII for acute intracerebral hemorrhage. N. Engl. J. Med. 358, 2127–2137. 10.1056/NEJMoa0707534 18480205

[B24] Mendelow A. D. Gregson B. A. Fernandes H. M. Murray G. D. Teasdale G. M. Hope D. T.. (2005). Early surgery versus initial conservative treatment in patients with spontaneous supratentorial intracerebral haematomas in the International Surgical Trial in Intracerebral Haemorrhage (STICH): a randomised trial. Lancet 365, 387–397. 10.1016/S0140-6736(05)70233-6 15680453

[B25] Mendelow A. D. Gregson B. A. Rowan E. N. Murray G. D. Gholkar A. Mitchell P. M.. (2013). Early surgery versus initial conservative treatment in patients with spontaneous supratentorial lobar intracerebral haematomas (STICH II): a randomised trial. Lancet 382, 397–408. 10.1016/S0140-6736(13)60986-1 23726393 PMC3906609

[B26] Qureshi A. I. Huang W. Lobanova I. Barsan W. G. Hanley D. F. Hsu C. Y.. (2020). Outcomes of intensive systolic blood pressure reduction in patients with intracerebral hemorrhage and excessively high initial systolic blood pressure: *post hoc* analysis of a randomized clinical trial. JAMA Neurol. 77, 1355–1365. 10.1001/jamaneurol.2020.3075 32897310 PMC7489424

[B27] Scaggiante J. Zhang X. Mocco J. Kellner C. P. (2018). Minimally invasive surgery for intracerebral hemorrhage. Stroke 49, 2612–2620. 10.1161/STROKEAHA.118.020688 30355183

[B28] Stumpo V. Staartjes V. E. Klukowska A. M. Golahmadi A. K. Gadjradj P. S. Schroder M. L.. (2021). Global adoption of robotic technology into neurosurgical practice and research. Neurosurg. Rev. 44, 2675–2687. 10.1007/s10143-020-01445-6 33252717 PMC8490223

[B29] Xia Z. Wu X. Li J. Liu Z. Chen F. Zhang L.. (2018). Minimally invasive surgery is superior to conventional craniotomy in patients with spontaneous supratentorial intracerebral hemorrhage: a systematic review and meta-analysis. World Neurosurg. 115, 266–273. 10.1016/j.wneu.2018.04.181 29730105

[B30] Xu X. Chen X. Li F. Zheng X. Wang Q. Sun G.. (2018). Effectiveness of endoscopic surgery for supratentorial hypertensive intracerebral hemorrhage: a comparison with craniotomy. J. Neurosurg. 128, 553–559. 10.3171/2016.10.JNS161589 28387618

[B31] Zhang K. Wei L. Zhou X. Yang B. Meng J. Wang P. (2022). Risk factors for poor outcomes of spontaneous supratentorial cerebral hemorrhage after surgery. J. Neurol. 269, 3015–3025. 10.1007/s00415-021-10888-w 34787693 PMC9120084

